# Breastfeeding support in Italian Pediatric Intensive Care Units: a nationwide survey on protocols, practices, and challenges

**DOI:** 10.1007/s00431-026-06896-5

**Published:** 2026-04-28

**Authors:** Daniela Morniroli, Marco Daverio, Riccardo Davanzo, Guglielmo Salvatori, Carlo Agostoni, Maria Lorella Giannì, Angela Amigoni, Gregorio Paolo Milani

**Affiliations:** 1 Department of Clinical Sciences and Community Health, University of Milan, Milan, Italy; 2 Pediatric Intensive Care Unit, Department of Women’s and Children’s Health, University Hospital of Padova, Padua, Italy; 3 Italian Society of Neonatology (SIN), Milan, Italy; 4 Maternal and Child Health Institute IRCCS “Burlo Garofolo”, Trieste, Italy; 5 Task Force On Breastfeeding, Ministry of Health, Rome, Italy; 6 Neonatal Intensive Care Unit, “Bambino Gesù” Children’s Hospital IRCCS, Rome, Italy; 7 Pediatric Unit, Fondazione IRCCS Ca’ Granda Ospedale Maggiore Policlinico, Milan, Italy; 8 Neonatal Intensive Care Unit, Fondazione IRCCS Ca’ Granda Ospedale Maggiore Policlinico, Milan, Italy

**Keywords:** Nationwide survey, Breastfeeding, Pediatric Intensive Care Units

## Abstract

Breastfeeding is recognized as the optimal source of nutrition in the first months of life. Pediatric Intensive Care Units (PICUs) play a crucial role in ensuring breastfeeding continuity for hospitalized infants in a critical care setting. However, despite the proven numerous benefits of breast milk in promoting the psychological well-being and short- and long-term health of hospitalized children, data on breastfeeding support in PICUs are sparse. This study aimed to assess the presence of written protocols and related clinical practices supporting breastfeeding in Italian PICUs. A cross-sectional survey with a 44-item questionnaire was conducted among the 26 Italian PICUs, collecting data on structural characteristics, breastfeeding policies, and parental involvement. Given the small sample size, Fisher’s exact tests, rather than chi-square tests, were used for comparisons. Logistic regression was performed to explore predictors of policy adoption. All the Italian PICUs participated. Results showed that only 27% of PICUs had written breastfeeding protocols, mainly covering infant feeding guidance, donor human milk use, and milk expression/storage, and 23% provided mandatory training for healthcare staff. While most units (96.1%) allowed at least one parent to remain with the child 24 h a day or to leave only for short periods, only 38.4% enabled maternal hospitalization in the same ward to support breastfeeding. Additionally, 57.6% actively promoted maintenance of maternal breast milk supply by providing breast pumps in the ward, while 61.5% considered offering donor human milk. Logistic regression analysis found no significant predictors of breastfeeding policy adoption, suggesting that institutional commitment and leadership engagement may be more influential than structural factors alone. These findings indicate that breastfeeding support in Italian PICUs is limited and inconsistent, underscoring the need for standardized national policies, written protocols, mandatory staff training, and improved infrastructure.

*Conclusion*: These findings indicate that breastfeeding support in Italian PICUs is limited and inconsistent, underscoring the need for standardized national policies, written protocols, mandatory staff training, and improved infrastructure.

**Table:** 

**What is known:** • *Breastfeeding offers important nutritional, immunological, and psychological benefits for critically ill infants, but intensive care hospitalization can compromise its continuity.* • *Although breastfeeding support has been widely studied in NICUs, evidence on policies and practices in PICUs is still very limited.*	
**What is new:** • *This nationwide Italian survey shows that breastfeeding support in PICUs remains heterogeneous, with limited use of formal protocols, staff training, and dedicated maternal support measures.* • *The absence of clear structural predictors suggests that local leadership and unit culture may play a central role in shaping breastfeeding practices.*

**Supplementary Information:**

The online version contains supplementary material available at 10.1007/s00431-026-06896-5.

## Introduction

The World Health Organization (WHO) recommends exclusive breastfeeding for the first 6 months of life, followed by continued breastfeeding alongside complementary foods up to 2 years or beyond [1]. Breastfeeding is indeed widely acknowledged as the gold standard for infant nutrition, offering a multitude of health benefits throughout infancy, particularly for critically ill infants. The immunological properties of human milk provide essential protection against infections, which is particularly beneficial for infants in intensive care settings [2]. Breast milk contains immunoglobulins (especially IgA), lactoferrin, lysozyme, and human milk oligosaccharides, all of which contribute to the infant’s developing immune system [3]. This protection is especially critical for infants admitted to intensive care units (ICUs), where they are exposed to hospital-acquired infections and complications in particular in those affected by pre-existing medical conditions or severe illness [4]. Human milk is often referred to as “personalized medicine” for infants due to its dynamic composition, which is also expressed through the exchange of immunoglobulins directed against pathogens that the dyad encounters in the environment. This is particularly relevant in ICU settings, where infants are often immunocompromised or recovering from severe illness. In addition, human milk contains anti-inflammatory cytokines and growth factors that promote tissue repair and modulate the immune response [5], which appears to be of fundamental importance to critically ill infants, often suffering from systemic inflammation due to infections or surgical procedures [6].

Breastfeeding is not just a source of nutrition and bioactive substances, but also a vital component of the mother-infant bond. It provides comfort, security, and emotional stability by lowering cortisol and heightening oxytocin levels in the dyad, fostering maternal-infant bonding and reducing stress for both mother and child, which is especially important in stressful environments like ICUs [7]. Infants admitted to ICUs are often separated from their mothers due to medical interventions, without considering that breastfeeding and skin-to-skin contact might mitigate the negative psychological effects of separation [7, 8]. Breastfeeding plays a crucial role in the psychological well-being of the mother. The ability to provide breast milk, even when direct breastfeeding is not possible, facilitates the active participation of mothers in their infant’s care. Studies suggest that mothers who are supported in breastfeeding or expressing milk for their hospitalized infants experience lower levels of postpartum depression and anxiety [9]. Despite these well-documented benefits, breastfeeding promotion in critical care settings faces several challenges, including maternal stress, medical interventions, hospital policies, and logistical barriers [8, 10].

While previous studies have examined breastfeeding support in Neonatal Intensive Care Units (NICUs) [11, 12], there is a lack of data specifically addressing breastfeeding in Pediatric Intensive Care Units (PICUs). Given the well-documented benefits of breastfeeding for infant health, immunity, and neurodevelopment, as well as the psychological advantages for both mothers and infants, it is essential to assess the extent to which Italian PICUs facilitate lactation and breastfeeding.

This study aims to assess organizational practices and the presence of written protocols supporting breastfeeding in Italian PICUs, identify barriers to its implementation, and determine whether institutional characteristics influence breastfeeding policy adoption.

## Methods

This study was conducted as a cross-sectional survey aimed at assessing the presence of written protocols, practices, and challenges related to breastfeeding support in Italian PICUs. The study was conducted according to the Declaration of Helsinki. The research utilized a structured questionnaire distributed to PICUs’ chief physicians to gather comprehensive data on unit characteristics and breastfeeding-related practices. The questionnaire was self-administered, implemented on LimeSurvey and disseminated electronically. Participating PICUs were identified through the national registry of intensive care centers in Italy (TIPNET Network [13]). Responses were collected over a designated period of 2 months to maximize participation. To encourage completion, reminders were sent periodically to non-respondents.

The questionnaire was developed based on previous studies evaluating breastfeeding practices in neonatal and pediatric intensive care settings [10, 14], and refined through expert consultation with pediatric and neonatal care professionals to ensure clarity, validity, and relevance. It comprised 44 items, aimed at exploring two primary aspects of breastfeeding support within Italian PICUs: (1) structural characteristics of the PICUs, including overall annual admissions and number of annual admissions of infants below 6 months of age; and (2) breastfeeding-related practices, including healthcare staff training, presence of written protocols (covering specific clinical conditions, handling and administration of human milk, use of donor milk and organizational or perioperative protocols); donor milk availability, parental involvement, and infrastructure for breastfeeding mothers. The full questionnaire is reported in the online supplementary material.

Participation in the survey was voluntary. Following data collection, all responses were compiled into a structured dataset for analysis. All statistical analyses were performed using IBM SPSS Statistics (version 29.0). Descriptive statistics were employed to summarize findings, including the frequency and distribution of key variables related to PICU characteristics and breastfeeding protocols. Categorical variables were presented as counts and percentages, while continuous variables were summarized using median values and interquartile range (IQRs). Given the small sample size and the low frequency counts observed in several contingency table cells, Fisher’s exact test was preferred. For comparisons, continuous variables such as annual PICU admissions were categorized into three levels based on their IQR. Parental presence was dichotomized as “frequent” (always or nearly always) versus “limited” (brief visits only), and perceived importance of breastfeeding by the PICU’s director was categorized as “no” (low importance) or “yes” (moderate or high importance). Additionally, logistic regression analysis was conducted to identify potential predictors of breastfeeding policy implementation in PICUs. The dependent variable was the presence of written breastfeeding protocols, while independent variables included annual PICU admissions, infant admissions, and mandatory staff training. A significance level of *p* < 0.05 was considered statistically significant.

## Results

### Survey response, descriptive analysis of PICU characteristics and breastfeeding policies

A total of 26 PICUs participated in the study, which comprised all the existing Italian PICUs. The median annual PICU admissions was 320 patients/year; median admissions involving infants younger than 6 months was 90 patients/year. Distribution of interquartile ranges were categorized as low, medium and high both for total admissions (low: < 200 (25°), median: 320, high: > 500 (75°), IQR: 300) and admissions of infants under 6 months (low: < 40 (25°), median: 90, high: > 200 (75°), IQR: 160). Breastfeeding support practices varied across units, with 27% (7 out of 26) reporting the availability of at least one written breastfeeding protocol for healthcare staff. Moreover, only 23% (6 out of 26) PICUs reported implementing mandatory breastfeeding training for healthcare personnel. The vast majority (25/26, 96%) of PICUs allowed at least one caregiver to stay with the infant 24 h a day or to be asked to leave only for justified short periods (i.e., during medical procedures or staff rounds), although only 12 out of 26 units (46.1%) allowed both parents to stay with their child at the same time. Only 19% (5/26) of PICUs provided educational materials regarding the importance of breastfeeding and human milk to parents, and 7 out of 26 (27%) provided mothers with guidance regarding the possibility of receiving breastfeeding support after discharge, through counseling by a health professional. Among those, only one unit employs a certified lactation consultant (International Board-Certified Lactation Consultant, IBCLC).

The large majority (84%) of PICUs acknowledged the importance of breastfeeding in the intensive care setting, but only 57% (15 out of 26) reported actively promoting maintenance of maternal breast milk supply by routinely providing breast pumps to mothers in their wards. Noticeably, 4 chief physicians stated that breastfeeding was of little importance in the PICU setting.

Only 38% (10/26) of PICUs reported allowing maternal hospitalization within the unit to facilitate breastfeeding, while 23% (6/26) allowed maternal hospitalization in a different ward, and more than half (15 out of 26, 57%) provided a more comfortable recliner or bed for breastfeeding mothers to rest and feed their infants.

Donor human milk was considered in 61% (16 out of 26) PICUs, but supply remained inconsistent due to supply limitations, mainly related to the high-priority allocation of donor milk to premature infants hospitalized in the NICU ward. Summary of PICU characteristics and breastfeeding protocols is reported in Table 1.

**Table 1:** Summary of PICUs characteristics and policies

Variable	*n*	25°–75° (IQR)
Annual PICU Admissions (Median)	320	200–500 (300)
Infant Admissions (< 6 months) (Median)	90	40–200 (160)
	Number of PICUs (n=26)	**Percentage (%)**
PICUs with Written Breastfeeding Protocols	7	26.9%
PICUs Providing mandatory Staff Training	6	23%
PICUs Considering Donor Human Milk	16	61.5%
PICUs Providing Breast Pumps to Mothers	15	57.6%
PICUs Allowing At Least One Parent in the Unit 24/7 or with the only exception of short periods (procedures or staff rounds)	25	96.1%

Fisher’s exact test did not reveal any significant association between the presence of at least one written breastfeeding protocol and total annual admissions (*p* = 1.000), and for admissions of infants under 6 months (*p* = 0.062). Similarly, there was no significant relationship between protocol presence and parental presence (*p* = 1.000), or between protocols and the perceived importance of breastfeeding by PICU’s director (*p* = 0.546).

No associations were found between the perceived importance of breastfeeding and either total admissions or infant admissions (*p* = 1.000 for both). In addition, mandatory training related to breastfeeding was not significantly associated with policy presence (*p* = 0.643), total admissions (*p* = 0.769), infant admissions (*p* = 0.549), or perceived importance of breastfeeding. In the logistic regression model, none of the predictors (total annual admissions, infant admissions under 6 months, or the presence of mandatory training) were associated with the presence of at least one written breastfeeding protocol (all *p* > 0.16) (Table 2).

**Table 2:** Binary logistic regression results

Variable	Coefficient (*β*)	Standard error	*p*-value	Exp(B)
Annual PICU admissions	0.004	0.003	0.163	1.004
Annual admissions of infants < 6 Months	–0.005	0.005	0.321	0.995
Mandatory training for healthcare staff	–0.468	1.249	0.708	0.626

## Discussion

This nationwide survey revealed that breastfeeding support in Italian PICUs is limited and inconsistently implemented, with fewer than one-third of units reporting written protocols or mandatory staff training, and significant gaps in infrastructure, maternal accommodation, and post-discharge support; moreover, no institutional or clinical factors were significantly associated with the presence of breastfeeding protocols, highlighting the likely role of local leadership and perceived importance of breastfeeding in shaping practices.

Previous research has highlighted several barriers to breastfeeding specifically referring to PICUs: many mothers struggle to continue lactation due to anxiety and the overwhelming experience of having a critically ill infant [15, 16]. In addition, some critically ill infants may not be able to breastfeed directly due to respiratory distress/symptoms, mechanical ventilation, sedation, or surgical recovery.

The importance of sustaining breastfeeding during hospitalization is underscored by the multidisciplinary working group of the Italian Ministry of Health [17]. The objective of the document *“Continuity of the mother-infant relationship and maintenance of breastfeeding during hospitalization”* is to ensure that breastfeeding mothers can remain with their child in the event of hospitalization, thereby reducing the risk of breastfeeding interruption. It aims to promote conditions that support maternal-infant proximity and the continuation of breastfeeding during hospital stays.

A retrospective study conducted in a Brazilian PICU assessed breastfeeding outcomes among hospitalized children between 2014 and 2016. The authors found that 13.6% of patients who were exclusively breastfed at admission were weaned by discharge, highlighting the negative impact of hospitalization on breastfeeding continuity. The study emphasizes the need for structured support strategies within PICUs to protect and sustain breastfeeding during critical care stays [18].

While the majority of PICUs chief physicians acknowledge the importance of breastfeeding, the data reveal considerable variability in policy implementation, lactation support services, and institutional infrastructure. This finding suggests that while hospitals may recognize the value of breastfeeding, this does not necessarily translate into greater institutional investment in breastfeeding support services. Although most of the available literature concerns the reality of NICUs, we hypothesized that the same issues can be found in PICUs.

Expanding lactation support services appears to be a crucial step. Only one PICU reported having a lactation consultant to guide breastfeeding mothers. Accordingly, Hookway et al. [19] conducted a systematic review on the challenges associated with breastfeeding medically complex children and reported that, although lactation consultants were available in neonatal settings, they were largely absent in pediatric wards, thereby complicating the continuation of breastfeeding. Conversely, when mothers did have access to a lactation consultant, they consistently reported this support to be beneficial. Increasing the availability of lactation consultants in PICUs, as recommended by Wakeham et al. [11] has been shown to improve breastfeeding rates by providing mothers with the guidance and encouragement needed to establish and maintain lactation. Within this complex scenario it has been highlighted that lactation professionals should work in close collaboration with the child’s primary healthcare team to ensure they are fully apprised of the treatment plan. They should also consult the child’s primary physician to determine whether any specific factors or considerations must be taken into account when developing an individualized lactation support program [7]. Only 6 out of 26 PICUs reported implementing mandatory training programs on breastfeeding support for their healthcare staff. This contrasts with international breastfeeding guidelines, which highlight the importance of professional lactation training for all involved healthcare staff in ICUs settings, in improving breastfeeding outcomes [20]. A study by Maastrup et al. [21] found that facilities offering mandatory breastfeeding training had significantly higher rates of breastfeeding at discharge. The limited training opportunities reported in Italian PICUs suggest that many healthcare providers may not have the necessary knowledge or confidence to effectively support breastfeeding mothers, potentially contributing to lower breastfeeding rates among critically ill infants. Despite these gaps, the study also highlights some positive findings. More than half of the PICUs actively promote the use of expressed breast milk by providing breast pumps, demonstrating institutional recognition of the benefits of human milk for critically ill infants. Donor human milk was considered as an alternative source, as reported by 61% of PICUs, but access remains inconsistent. Expanding access to donor milk in Italian PICUs could therefore have significant benefits for infant health, particularly for infants whose clinical condition would benefit from the specific properties of human milk, including its trophic factors, such as post-surgical neonates, or its digestibility, as in the case of infants with cardiovascular disorders.

Parental involvement is another key factor influencing breastfeeding success, yet this study found that only 38% of PICUs allowed maternal hospitalization within the same unit to facilitate breastfeeding. Research by Brødsgaard et al. [22] in a NICU setting highlights the critical role of parental presence in facilitating breastfeeding, as mothers who have continuous physical contact with their infants are more likely to maintain milk production and continue breastfeeding. The limited parental access reported in some Italian PICUs may hinder mothers from establishing a successful breastfeeding routine, further emphasizing the need for more family-centered care models. Additionally, 42% of PICUs do not provide designated breastfeeding-friendly spaces, which could further discourage breastfeeding within the hospital setting.

Improving infrastructure to support breastfeeding mothers should also be a priority. Providing dedicated breastfeeding rooms within PICUs, as well as ensuring that mothers have access to hospital-grade breast pumps, can facilitate lactation even when direct breastfeeding is not possible. Research from Van Dellen et al. [23] on the effects of comfort and quality of designated lactation rooms in a work setting has demonstrated that better breastfeeding spaces report higher rates of maternal milk expression, emphasizing the importance of creating a supportive physical environment for breastfeeding.

One of the most critical findings of this study is that only 27% of Italian PICUs interviewed reported having written breastfeeding protocols in place. This means less than one third of the surveyed units have clear guidelines, which could lead to inconsistencies in the way breastfeeding is encouraged and supported. Similar challenges have been identified in NICUs worldwide, explaining why the implementation of the first of the Ten Steps to Successful Breastfeeding is reflected in breastfeeding rate at discharge [24]. The lack of formalized protocols may contribute to uncertainty among healthcare staff regarding best practices for lactation support, further exacerbating the challenges faced by mothers attempting to breastfeed in intensive care settings.

Our study reveals no significant predictor of breastfeeding policy adoption. Annual PICU admissions showed non-significant correlation with policy presence, indicating that larger PICUs were not necessarily more likely to have breastfeeding protocols. Interestingly, mandatory staff training was not significantly associated with the presence of written breastfeeding protocols, despite its recognized importance in previous research [10]. This could indicate that even when training is available, other factors such as hospital culture, administrative support, and funding play a larger role in shaping breastfeeding practices. These findings indicate that hospital size and training alone do not predict the presence of written breastfeeding protocols, suggesting that leadership engagement and hospital culture may play a more influential role.

This study has some limitations that should be considered when interpreting the results. First, although the sample includes all PICUs in Italy, the absolute number of centers (*n* = 26) is relatively small, which may reduce statistical power and limit the detection of subtle associations in multivariable analyses. Additionally, the data were self-reported by PICU representatives, which introduces the possibility of reporting bias or subjective interpretation, particularly in the assessment of training activities, protocol presence, and policy implementation. The study is also geographically limited to Italy, and while multicenter in nature, the findings may not be generalizable to PICUs in other countries with different healthcare systems, breastfeeding cultures, or policy frameworks.

## Conclusions

This study provides an initial overview of breastfeeding-related practices within Italian PICUs. Despite including all national units, no statistically significant associations were found between written protocols presence and institutional factors such as admission volume, infant admissions, or training initiatives. These findings highlight the complexity of policy implementation in critical care settings and underscore the need for further research, potentially on a larger, international scale, to better understand the structural and cultural factors that support breastfeeding in pediatric intensive care environments.

The inconsistencies identified in this study highlight the urgent need for standardized national policies on breastfeeding support in PICUs. Countries that have implemented comprehensive breastfeeding policies have reported higher rates of breastfeeding initiation and continuation in intensive care settings [21]. The Italian healthcare system could benefit from adopting similar approaches, including routine staff training and hospital-wide breastfeeding promotion initiatives.

It also appears valuable to promote breastfeeding culture during scientific and educational events organized by societies focused on pediatric intensive care. Creating dedicated sessions for breastfeeding-related topics, in full compliance with the International Code of Marketing of Breast-milk Substitutes and independent of commercial milk formula interests [25], may help foster progressive awareness and engagement within the PICU community.

## Supplementary Information

Below is the link to the electronic supplementary material.

ESM 1 (DOCX 17.8 KB)

## Data Availability

This study was partially founded by Italian Ministry of Health, Current Research IRCCS.
