# Supplementary material for: Breastfeeding support in Italian Pediatric Intensive Care Units: a nationwide survey on protocols, practices, and challenges
Source: Eur J Pediatr. 2026 Apr 28;185(5):312. doi: 10.1007/s00431-026-06896-5 (PMC13124803; doi:10.1007/s00431-026-06896-5)
Supplement: Supplementary file 1 — (DOCX 17.8 KB) [file 431_2026_6896_MOESM1_ESM.docx]

**Supplementary Material**

**S1. Questionnaire administered to Italian PICUs**

# Pediatric Intensive Care Unit – Breastfeeding Practices Survey

## Section 1: Structural Characteristics of the PICUs

1. What is the name of the hospital/center and in which city is it located?

2. What is the average annual number of admissions to your pediatric intensive care unit (PICU)?

3. What is the average annual number of admissions of infants under 6 months of age?

## Section 2: Breastfeeding-Related Practices

4. Is mandatory training on the promotion and support of breastfeeding during hospitalization provided to healthcare personnel in your unit?

5. How frequently is this training offered?

6. Are written protocols available to medical and nursing staff regarding breastfeeding in your PICU?

7. Are there written protocols for breastfeeding support for infants with tongue-tie?

8. Are there written protocols for breastfeeding support for infants with cleft lip/palate?

9. Are there written protocols for breastfeeding support for infants with congenital heart disease?

10. Are there written protocols for breastfeeding support for infants with neurological disabilities?

11. Are there written protocols for the expression, storage, and administration of human milk?

12. Are there written protocols for the use of donor human milk?

13. Are there written protocols for pre-anesthesia or preoperative fasting in breastfed vs. formula-fed infants?

14. Are there written protocols for any other condition (including other pathologies or situations)?

15. If 'Other condition', please specify for which condition written protocols are available:

16. Is breastfeeding-related informational material routinely provided to parents in your PICU?

17. Is there informational material about breastfeeding for infants with tongue-tie?

18. Is there informational material about breastfeeding for infants with cleft lip/palate?

19. Is there informational material about breastfeeding for infants with congenital heart disease?

20. Is there informational material about breastfeeding for infants with neurological disabilities?

21. Is there informational material about expression, storage, and administration of human milk?

22. Is there informational material about the use of donor human milk?

23. Is there informational material about pre-anesthesia or preoperative fasting in breastfed vs. formula-fed infants?

24. Is there informational material about any other condition (including other pathologies or situations)?

25. During hospitalization, is one parent at a time allowed to stay with their child?

26. Are parents asked to leave or not allowed to enter during medical rounds?

27. Are parents asked to leave or not allowed to enter during major invasive procedures (e.g., CVC placement, drain insertion)?

28. Are parents asked to leave or not allowed to enter during minor procedures (e.g., venous or capillary blood draws)?

29. Are parents asked to leave or not allowed to enter during specialist consultations?

30. Are parents asked to leave or not allowed to enter during nursing and medical shift handovers?

31. Are parents asked to leave or not allowed to enter during the night?

32. Are both parents allowed to be present with their child at the same time (co-presence)?

33. Does your hospital allow mothers to be admitted to the PICU to continue breastfeeding while their infant is hospitalized?

34. Is it routine to suggest that mothers express and refrigerate their breast milk when the baby cannot feed directly at the breast?

35. Are breast pumps provided to mothers whose baby is unable to feed?

36. Are bottles available for the collection and administration of expressed breast milk?

37. Does the breastfeeding mother have access to a bed or recliner in the room to rest and breastfeed during her child’s stay?

38. At the time of discharge, are mothers informed about the possibility of receiving support from peer counselors or breastfeeding support groups?

39. Do you consider offering donor human milk to patients admitted to your PICU?

40. Is donor milk reserved only for highly preterm infants in the neonatal intensive care unit (NICU)?

41. Is it because there is no human milk bank available at your hospital?

42. Is it because your hospital has never requested donor milk from a regional milk bank?

43. Have you requested donor milk from a regional milk bank but were unable to obtain it?

44. Do you believe that breastfeeding is a relevant topic in the context of a pediatric intensive care unit?
